# Comparative Analysis of the Fecal Proteome in Two Canine Breeds: Dalmatians and Weimaraners

**DOI:** 10.3390/ijms26178247

**Published:** 2025-08-25

**Authors:** Matteo Cerquetella, Francesco Pinnella, Rachele Morazzini, Giacomo Rossi, Andrea Marchegiani, Alessandra Gavazza, Sara Mangiaterra, Alessandro Di Cerbo, Daniela Sorio, Jessica Brandi, Daniela Cecconi, Silvia Vincenzetti

**Affiliations:** 1 School of Biosciences and Veterinary Medicine, University of Camerino, Via Circonvallazione 93/95, 62024 Matelica, MC, Italy; 2 Futuravet Veterinary Referral Center, 62029 Tolentino, MC, Italy; 3 Centre for Technological Platforms (CPT), University of Verona, Piazzale L.A. Scuro 10, 37134 Verona, VR, Italy; 4 Department of Biotechnology, University of Verona, Strada le Grazie 15, 37134 Verona, VR, Italy

**Keywords:** dog, fecal proteome, biomarker, breed influence, pathogenesis

## Abstract

The analysis of proteins in stool samples can significantly enhance the study of mammalian physiology and disease. In this study, we investigated the fecal proteome of clinically healthy dogs (n = 26) by a label-free proteomics approach to evaluate the impact of breed differences. The dogs were divided into two groups (n = 13 each) based on their breed, specifically Weimaraner and Dalmatian, the former known for their possible susceptibility to gastrointestinal disease. Quantitative and qualitative differences between the two experimental groups were identified based on analyses performed on pooled biological samples. The overall fecal proteome profile comprised 58 proteins, of which 37 were common, while comparative proteomics analysis detected 15 proteins with different abundances. Notably, the fecal proteome of Weimaraners showed an over-representation of proteins such as pantetheinase, which promotes inflammatory reactions; ferritin heavy chain and hemoglobin, possibly associated with gut ulceration and/or rectal bleeding typical of IBD; and anionic trypsin, implicated in inflammatory bowel disease. Notably, in Dalmatians, despite the absence of specific predispositions, some proteins associated with chronic enteropathy (e.g., carboxypeptidase B and serine protease 1) were also over-represented. Additionally, some proteins linked to breed variation included enzymes associated with “protein digestion and absorption” and “glycolysis and gluconeogenesis”. These findings suggest, for the first time, that the variable breed is a factor that may potentially influence the fecal proteome in dogs.

## 1. Introduction

The study of the fecal proteome has recently been introduced in canine and feline medicine, and it is suggested as a useful tool to evaluate and better understand the gastrointestinal (GI) environment in both healthy and diseased subjects [1,2,3,4,5,6]. Proteomics is a strategy to identify new biomarkers useful for diagnostic, prognostic, and monitoring purposes [7,8]. Unfortunately, no data are available to determine whether physiological variables, such as breed, can influence the composition of the fecal proteome in dogs.

It is known that for different breeds, there is a predisposition to GI diseases. Dalmatian is a canine breed for which no specific breed predisposition to GI diseases has been reported (despite this breed having a metabolic disorder in the metabolism of purines, forming urate uroliths due to hyperuricemia and hyperuricuria caused by dysfunction in the metabolism of purines, reducing allantoin excretion) [9]. At the same time, Weimaraner is considered susceptible to developing inflammatory bowel disease (IBD) (although geographical/environmental factors have also been hypothesized) [10,11].

The present study aimed to investigate, for the first time, the fecal proteome of clinically healthy dogs, divided by breed, to evaluate whether this physiological variable may influence the GI proteome and, if so, to what extent. We assumed that the Weimaraner breed may represent a variable possibly associated with changes in the fecal proteome.

## 2. Results

### 2.1. General Profile of the Fecal Proteome

The knowledge of the proteome extracted from fecal samples can offer valuable insights into the adaptive regulation of intricate physiological processes linked to nutrient digestion and the maintenance of gut microbiome homeostasis. We hypothesized that the canine breed could influence the fecal proteome, which comprises proteins discharged into the gastrointestinal tract, including enzymes, mucosal proteins, secretory immune proteins, and proteins released from epithelial cells. Therefore, in this study, LC-MS analysis was conducted on stool samples collected from healthy dogs of two different breeds (WD, DD).

**Figure 1:**
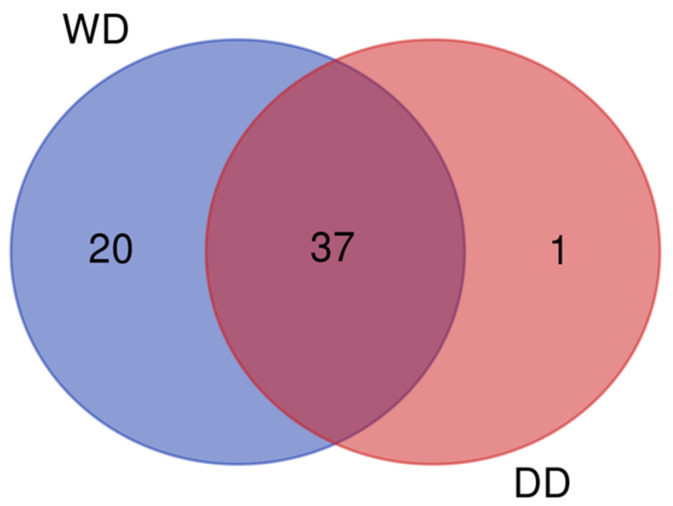
Venn diagram showing the overlap between the proteins identified in the two groups. Data shown were obtained from pooled samples (n = 13 per group). WD = Weimaraner dogs, DD = Dalmatian dogs.

To specifically identify peptides from dogs, spectra were compared to those of “*Canis lupus familiaris*” peptides in the Uniprot database, requiring at least a unique peptide for each protein identification. Considering that proteins from food and the microbiota may also be present, the searches were limited to the dog proteome database, which aligned with the aim of this work. The list of identified proteins for each sample is reported in Table 1. In particular, a total of 58 different proteins were identified in the feces from the two groups of dogs: 57 in WD and 38 in DD. The Venn diagram in Figure 1 shows the overlap between proteins in the different groups.

**Figure 2:**
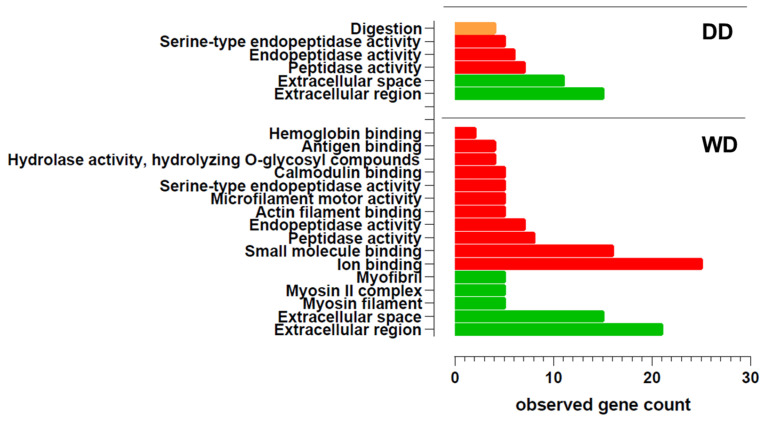
Gene Ontology (GO) enrichment analysis of proteins that were present in the fecal proteome of dogs (*Canis lupus familiaris*) in two different breeds: Dalmatian (DD) and Weimaraner (WD). Highly significant GO terms for biological process (GO:BP, orange), molecular function (GO:MF, red), and cellular component (GO:CC, green) are presented. Data shown were obtained from pooled samples (n = 13 per group).

Gene ontology enrichment analysis (Figure 2 and Supplementary Table S1) revealed that both groups share the same main cellular components. In both breeds, the fecal proteome is characterized by proteins localized at the extracellular level. Additionally, in the WD breed, muscle-related proteins, such as myosins and myofibrils, were also detected. Regarding molecular function, proteins with peptidase and endopeptidase activity were identified in the feces of both breeds. Furthermore, the WD group exhibited fecal proteins with various binding activities (e.g., ion binding, small molecule binding, calmodulin binding, hemoglobin binding). Interestingly, only in the DD breed, the GO analysis revealed an enrichment in the biological process of digestion.

### 2.2. Comparative Fecal Proteomics Analysis Between Dalmatian and Weimaraner

**Figure 3:**
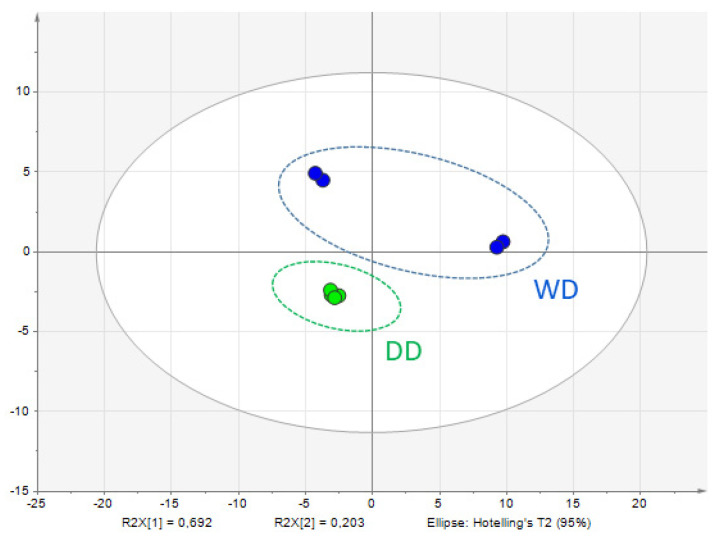
Score plot of PCA to overview the classification trend of proteome profiles of WD (blue circles) and DD (green circles) samples. Data shown were obtained from pooled samples (n = 13 per group). WD = Weimaraner dogs, DD = Dalmatian dogs.

Subsequently, the protein profiles of the feces were investigated using a label-free quantitative proteomic approach. Principal Component Analysis (PCA) was performed to explore the multivariate structure of the data. As illustrated in Figure 3, the first and second principal components accounted for 69.2% and 20.3% of the total variance, respectively, indicating notable variation among the sample groups. PCA findings should be considered exploratory. Nonetheless, the observed separation supports the hypothesis that significant proteomic alterations are present and may be biologically relevant.

**Figure 4:**
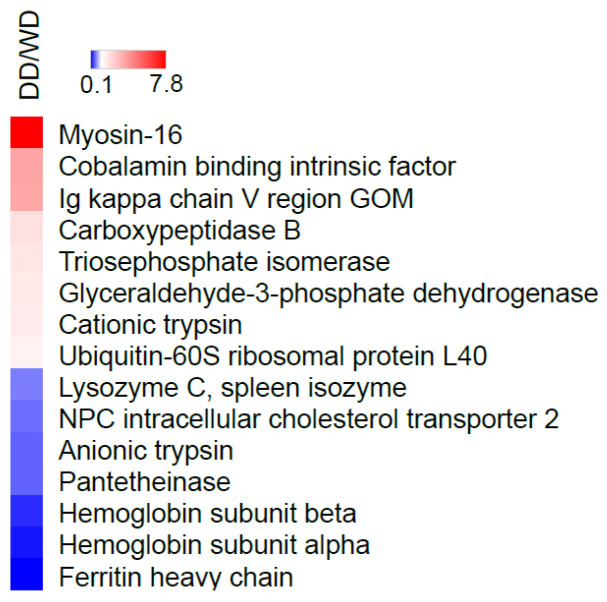
Heatmap showing regulated proteins (blue = downregulated, oxblood/red = upregulated) in the comparative analysis of DDs vs. WDs. WDs = Weimaraner dogs, DDs = Dalmatian dogs.

A comparison of the quantitative protein levels between the samples showed 15 proteins that were significantly dysregulated in the stool of the Dalmatian dogs compared to the Weimaraner dogs (Supplementary Table S2). These proteins, which showed different abundance levels in the feces of dogs, are summarized in Table 2 and in Figure 4. Among them, eight proteins were identified as more abundant in the stool of the Dalmatian dogs, and seven proteins were more abundant in the stool of the Weimaraner dogs.

**Figure 5:**
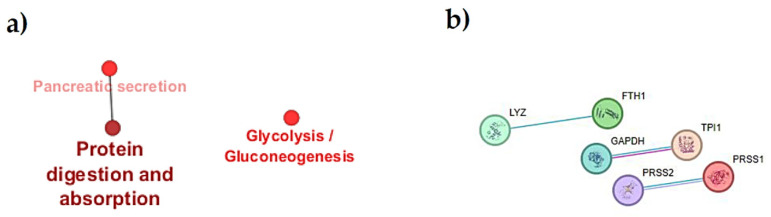
Pathways and protein network. (**a**) Analysis of enriched KEGG pathways of dysregulated fecal proteins related to breed. The node colors depict the enrichment significance (ranging from red = *p*-value < 0.05 to dark red = *p*-value < 0.005 and dark brown = *p*-value < 0.0005). (**b**) Interaction analysis of dysregulated fecal proteins. The circles represent the identified proteins; the edges represent protein–protein interactions (blue lines = known interactions from curated databases, pink lines = experimentally determined interactions). LYZ = lysozyme C, spleen isozyme, FTH1 = ferritin heavy chain, GAPDH = glyceraldehyde-3-phosphate dehydrogenase, TPI1 = triosephosphate isomerase, PRSS2 = anionic trypsin, PRSS1 = serine protease 1. Data shown were obtained from pooled samples (n = 13 per group).

Then, to uncover signaling pathways and interaction networks involving these proteins, we conducted a bioinformatics analysis on those with significantly altered abundance. Enrichment analysis identified statistically significant enriched KEGG pathways (*p* < 0.05) for proteins with a different abundance in the stool of the two groups of dogs (i.e., Dalmatian or Weimaraner): they included “glycolysis/gluconeogenesis” (with GAPDH and TPI1 proteins implicated) as well as “Protein digestion and absorption” (involving CPB1 and PRSS2) (Figure 5a and Table 3).

Moreover, the network interaction between proteins shed into feces was analyzed using the STRING database to detect which could interact within established complexes or have functional relationships. As shown in Figure 5b, the protein–protein interaction (PPI) network derived from proteins with different abundances in the dogs’ stool related to breed exhibited a low average node degree, as indicated by only three pairs of interacting proteins. Among these, the interacting GAPDH and TPI1 enzymes were detected, further suggesting that glycolysis and gluconeogenesis are key pathways that may distinguish the fecal proteome of Dalmatians from that of Weimaraner dogs.

## 3. Discussion

The present study described and compared the fecal proteome in two groups of clinically healthy dogs divided by breed (Dalmatian and Weimaraner).

The study of the fecal proteome has great potential in understanding the gastrointestinal environment in both health and disease. In humans, in fact, it has been seen that the analysis of the fecal proteome may help in distinguishing healthy controls from patients with Crohn’s Disease and Ulcerative Colitis, as well as from patients with Gastric Carcinoma. Parallelly, it may allow the identification of specific markers potentially capable of differentiating between the various pathologies (e.g., increased sucrose-isomaltase enzyme in Crohn’s Disease vs. Ulcerative Colitis) [12].

Although difficult to interpret, the total number of proteins highlighted in the two different groups of the present study is noteworthy; indeed, in the WD group, 57 proteins were identified, while in the DD group, only 38 were found. To hypothesize a reason for this finding, we could speculate that some breeds shed more or less protein in their feces than others, but this is a consideration that certainly needs to be confirmed in future studies. Notably, the WD fecal proteome seemed to be characterized by 20 specific proteins not identified in the feces of the DD group. This finding was interesting when analyzed in light of the initial hypothesis, which suggested that the Weimaraner breed may represent a variable associated with a fecal proteome different from the “normal” one. On the other hand, the fecal protein profile of the DD group was characterized by only one protein (WAP four-disulfide core domain protein 2). Finally, the fact that the greatest number of proteins (n = 37) was in common between the two groups reinforces the idea of a core of proteins excreted into feces across all canine patients, regardless of the variables considered. Interestingly, examining Table 1 reveals the presence of a cluster of different myosin classes (myosins 2, 4, 7, 13, and 16) that are specifically found in the feces of WDs. It is known from the literature that the intestinal brush border contains at least 14 myosin members, of which the most studied are myosin 1a, non-muscle myosin 2c, myosin 5b, myosin 6, and myosin 7b. The role of these myosin isoforms is to regulate the assembly, morphology, and function of microvilli [13,14]. The microvillar membrane is indeed maintained due to a coordinated balance between myosins exerting opposite forces. In particular, among the myosins found in the feces of WDs, myosin 2 manages several aspects of microvilli architecture and mobility. In contrast, myosin 7 is an important component of a complex that maintains the mechanical tension across the heterophilic cadherin links between microvilli [14]. It is well documented in the literature that apical microvilli are perturbed in several intestinal diseases, including microvillus inclusion disease, Crohn’s Disease, and infections with enteric pathogens [15,16]. Other authors have indicated that myosin light chains 9, 12a, and 12b are involved in the pathogenesis of IBD and suggested using them as new therapeutic targets for patients suffering from IBD [17]. In light of these considerations, the presence of myosins in the feces could be associated with a perturbation of the microvilli following an intestinal pathology. Regarding the quantitative proteomic results, notable differences emerged when comparing the two groups. In total, 15 different proteins were found to be dysregulated in the feces of the 26 dogs included in this study. In particular, comparable numbers of proteins were identified as more abundant in the fecal proteome of Dalmatian dogs (eight proteins) and Weimaraner dogs (seven proteins) (Table 2). Among the proteins dysregulated and overrepresented in Weimaraner dogs, NPC intracellular cholesterol transporter 2 (NPC2) was of interest since it is involved in the export of cholesterol from lysosomes along with NPC1. These two proteins possess sterol-binding domains. NPC2 binds cholesterol in the lumen and then transfers it directly to NPC1; the latter, in turn, seems to be involved in the transport of cholesterol into and/or across the lysosomal membrane [18,19]. Finally, cholesterol is delivered to endosomes, the ER, the Golgi, and the plasma membrane with a largely unknown mechanism. NCP2 is expressed in the nervous and respiratory systems, as well as the stomach, and is located in lysosomes. A polymorphism in the *NPC1* or *NPC2* gene is associated with lysosomal cholesterol accumulation and, consequently, with the Niemann–Pick disease type C, a human neurodegenerative lysosomal lipid storage disorder associated with the early onset of Crohn’s Disease [20,21,22]. Pantetheinase, also found in higher levels in the fecal proteome of WDs than DDs, is an enzyme that breaks down pantetheine, an intermediate compound in the coenzyme A degradation pathway, into pantothenate (vitamin B5) and cysteamine. Studies suggest that it plays an active role in promoting inflammation since the product cysteamine is a molecule able to inhibit the enzyme γ-glutamylcysteine synthase, which is involved in synthesizing the redox stress regulator GSH. When the level of cysteamine is high in tissues, there is an increase in oxidative stress and, consequently, inflammation [23]. It has been shown that in the gut, the induction of this enzyme reflects a local adaptation to metabolic or oxidative stress [24]. Giessner and co-workers [25] identified Vnn1, a pantetheinase, as a tumor suppressor for the development of aggressive forms of soft tissue sarcomas. These authors found that the pantothenate produced by enzymatic activity increases the amount of CoA, thereby enhancing mitochondrial activity. In contrast, the other product, cysteamine, prevents the Warburg effect, a characteristic of the most aggressive tumors [26]. More recently, Millet and co-workers [27] found that Vnn1 is overexpressed in inflamed colonocytes, which correlates with the severity of IBD. The greatest activity of this enzyme in the colon is cytoprotective since it leads to the regeneration of CoA and the production of short-chain fatty acids (especially butyrate) by the microbiota. However, in severe IBD, the lack of substrates could impair the enzymatic activity of pantetheinase, even when overexpressed, thereby compromising its cytoprotective effect. Ferritin heavy chain was also of interest as ferritin heavy chain 1 is an anti-oxidative gene, and its upregulation was observed in a mouse colitis model fed with bioactive peptides with antioxidant properties compared to controls [28]. Additionally, ferritin heavy chain, as well as hemoglobin subunits alpha and beta (the other two proteins found to be more abundant in the stool of WDs), may be related to gut ulceration and/or rectal bleeding, which is typical of IBD in humans as well [29]. The lysozyme C spleen isozyme, which was more abundant in WDs than in DDs, was also described in a previous study, carried out with the same methods as the present one, on both healthy patients and dogs affected by different hepatobiliary disorders. In that study, the lysozyme C spleen isozyme was overrepresented in the feces of dogs showing clinical, ultrasonographic, and/or laboratory evidence of different hepatobiliary dysfunctions than in chronic cases [6]. Finally, anionic trypsin is also of interest, as protease activity was found to be increased in a study on human IBD patients, among whom the serine protease family was found to be the most active, particularly trypsin [30].

Regarding proteins that were more abundant in the DD group, those of greater interest were cobalamin-binding intrinsic factor, carboxypeptidase B, and serine protease 1. The first one is a glycoprotein produced by the parietal cells located at the gastric body and fundus, and it is necessary for the transportation and later absorption of vitamin B12 in the distal ileum of the small intestine [31]. It should be emphasized that hypocobalaminemia is commonly associated with chronic enteropathies in dogs [32]. The same protein was identified in a previous study [6], which found it to be more abundant in the feces of chronic cases than in dogs with various hepatobiliary disorders. Carboxypeptidase B is involved in the coagulation and fibrinolysis processes that regulate them [33]. However, it was found to be significantly more abundant in the feces of dogs suffering from chronic enteropathy than in healthy controls [4]. It was also found to be increased in the feces of diseased dogs (liver diseases) compared to controls in a methodologically similar previous study of the same research group [6]. Concerning the last study [6], serine protease 1 (also known as cationic trypsinogen) behaved the same, and it was also found to be increased in DDs in this study. In addition, as reported above, the anionic form was also found to be increased in WDs, therefore attributing a contradictory and hardly justifiable meaning to the finding in both groups.

Considering the above, it is plausible to speculate that the greater (but not exclusive) presence of proteins associated with GI diseases in the WD fecal proteome may contribute to, or be a consequence of, the possible WD predisposition to inflammatory bowel disease.

To better evaluate the effects of the Weimaraner dog breed on the fecal proteome, a bioinformatic analysis was performed. Overall, a significant link between two pathways, including “protein digestion and absorption” and “glycolysis and gluconeogenesis”, was detected for four proteins that had a lower abundance in the stool of WDs as compared to DDs. The finding that two proteases implicated in “protein digestion and absorption”, i.e., serine protease 1 and carboxypeptidase B, were at low abundances in the feces of WDs is somewhat surprising and will require further investigation. Indeed, fecal protease activities were higher in IBD human fecal samples than in healthy controls [30], and the carboxypeptidase B enzyme was found to be abundant in the fecal proteome of dogs affected by enteropathies. However, although these data may seem discordant, it should be emphasized that the study included various canine breeds and did not focus only on WDs, as in our investigation [4]. Moreover, the “glycolysis/gluconeogenesis” pathway seems more difficult to explain, even if it was associated with colonic adenocarcinoma, with an underlying mechanism that needs to be fully clarified [34].

This study presents some limitations that should be acknowledged. First, the use of a pooling strategy without biological replication restricts the ability to assess inter-individual variability and may obscure subject-specific molecular signatures. Second, potential confounding factors, such as age and sex, were not controlled for, which could influence the observed proteomic profiles. Third, the analysis was conducted at a single time point, limiting insights into temporal dynamics. Fourth, the absence of microbiome data prevents integrated multi-omics interpretations that could further elucidate host–microbiota interactions. Moreover, no multiple testing correction was applied to the *p*-values of stool proteins with different abundance levels, which implies that 2–3 of them could potentially represent false positives. Finally, the lack of orthogonal validation techniques, such as quantitative PCR (qPCR) or Western blotting, limits the confirmation of differential abundance for key proteins identified through label-free proteomic analysis.

Future developments of the present study concern the possibility of carrying out the same evaluations on individual subjects. It will also be interesting to expand this study to a greater number of patients and further breeds for which, for example, different predispositions are described. Furthermore, transcriptomic profiling of fecal samples could offer complementary insights to reinforce and validate the proteomic findings. Integrating multi-omics approaches has been shown to enhance the resolution and functional interpretation of molecular signatures.

## 4. Materials and Methods

### 4.1. Dogs Included in This Study

In the present study, 26 clinically healthy dogs free from any known acute or chronic disease and not receiving any medication were enrolled (Table 4). Furthermore, they had not presented any episodes of diarrhea in the last month or any other clinical signs of other diseases in the same period. All the subjects were regularly subjected to periodic hematological evaluations and treatments for ecto- and endo-parasites. All the dogs were fed a maintenance kibble diet. Data concerning the age of each group were first checked for normality tests using the D’Agostino–Pearson normality test. The dogs were divided into two groups of 13 animals each, according to breed: Weimaraner dogs [(WDs; 46% males and 54% females, age: 5.5 + 2.9 years (mean + SD)] and Dalmatian dogs [(DDs; 38% males and 62% females, age: 7.5 + 3.7 years (mean + SD)].

### 4.2. Samplings and Protein Extraction, Digestion, and Purification

As previously reported in the literature [6], naturally voided fecal samples, immediately after evacuation, were frozen at −20° C until use. From each sample, 2 g of stool was withdrawn and pooled to obtain 20 g of feces per group. Each pooled sample (WDs and DDs) was resuspended in 60 mL of phosphate-buffered saline (PBS), supplemented with a protease inhibitor cocktail (1:100 final dilution, Sigma-Aldrich, Saint Louis, MO, USA). Given that the aim of this study was to identify broad molecular patterns and potential biomarkers across populations, a pooling strategy was adopted. This approach minimizes biological variability and technical noise, thereby enhancing the detection of consistent molecular signals by averaging out individual-specific fluctuations. Moreover, pooling the samples allowed for greater cost-efficiency and resource optimization.

The entire protein extraction procedure was carried out on ice and, as previously published [5,35], ensured the integrity of the extracted proteins. The obtained solution was stirred for one hour on ice and was subsequently centrifuged at 10,000× *g* for 20 min. Each resulting supernatant was subjected to three filtration steps: in the first one, filter paper was used (three times), and in the second and third steps, 0.45 mm (one time) and 0.20 mm (one time) sterile membranes (Whatman, Maidstone, UK) were used, respectively. Ammonium sulfate at 90% was slowly added to each filtrate while gently stirring for 30 min, maintaining the sample on ice. The samples were subsequently centrifuged at 27,000× *g* for 30 min.

After centrifugation, the precipitate was recovered and resuspended in 1 mL of PBS. Afterwards, the total protein content in each solution thus obtained was determined using the Bradford method [36]. Proteins (0.1 mg) were extracted from each solution using the EasyPep™ Mini MS Sample Prep Kit (Thermo Fisher Scientific, Rockford, IL, USA) following the manufacturer’s instructions. Protein concentrations were determined using the Pierce™ BCA Protein Assay Kit (Thermo Fisher Scientific, Waltham, MA, USA). Subsequently, 50 µg of total protein was enzymatically digested with Trypsin/Lys-C Protease mix (provided by the kit) at an enzyme-to-protein ratio of 1:50, under continuous shaking at 37 °C for 3 h. After digestion, the samples were dried using a vacuum centrifuge concentrator. Subsequent LC-MS analysis was performed on each pellet containing 0.1 mg of digested proteins, resuspended in 100 mL of 0.1% formic acid.

### 4.3. LC-MS/MS Analysis

As described previously [6], experiments were conducted using an Ultimate 3000 nanoUPLC system (Thermo Fisher Scientific, Waltham, MA, USA) paired with an Orbitrap Fusion Lumos Tribrid mass spectrometer (Thermo Fisher Scientific, Waltham, MA, USA). To separate peptides via nanoUHPLC, 2 μL of the sample (equivalent to 1 μg of peptides) was loaded onto the analytical column (Easy-Spray PepMap RSLC C18, 2 µm, 500 × 0.075 mm, Thermo Fisher Scientific, Waltham, MA, USA). Peptides were eluted using a gradient of mobile phases (i.e., 0.1% formic acid and 0.1% formic acid plus acetonitrile) for 120 min at a 300 nL/min flow rate. Ionization was performed via a nanoESI source in positive mode, with a 1.5 kV voltage and a capillary temperature of 275 °C. MS1 spectra were captured by the Orbitrap in data-dependent acquisition mode over a 375 *m*/*z* to 1500 *m*/*z* range, at a resolution of 120,000 (at 200 *m*/*z*), using standard AGC settings. MS2 spectra were recorded by the Orbitrap at a 50,000 resolution (at 200 *m*/*z*), isolating precursor ions with intensities above 3 × 10^4^ and charges between +2 and +5 within a 2.0 Da window. Fragmentation was performed via high-energy C-trap dissociation with a normalized collision energy of 30%. A 30 s dynamic exclusion window was used to avoid redundant fragmentation of identical ions. All experiments were performed in quadruplicate for each condition.

### 4.4. Data Analysis

Protein detection and label-free quantification were carried out by Proteome Discoverer software (v2.5). Protein identification utilized the Sequest HT search algorithm with the following parameters: carbamidomethylation (C) as a fixed modification, and oxidation (M) and acetylation at the protein N-terminus as variable modifications. The database employed was Uniprot version release 2025_03 (*Canis lupus familiaris entries*), with trypsin set as the enzyme, and up to two missed cleavages were permitted, along with the inclusion of common contaminants. Peptides with a minimum length of 5 amino acids were considered, with mass tolerances of 10 ppm for precursor ions and 0.02 Da for MS/MS data in the Orbitrap. A false discovery rate (FDR) of 1% was applied for both proteins and peptides to ensure reliability of the identifications. Label-free quantification was performed using a minimum of two unique peptides for protein abundance estimation. Statistical significance was assessed using a two-tailed Student’s *t*-test for *p*-value calculation, performed in Microsoft Excel v.2024. Proteins with a *p*-value < 0.05 and a fold change (FC) of ±1.5 were considered significantly modulated. The mass spectrometry proteomics data were deposited to the ProteomeXchange Consortium via the PRIDE [37] partner repository with the dataset identifier PXD066651.

### 4.5. Bioinformatics Analysis of Omics Data

The proteomic data underwent bioinformatic analysis using established methods [38]. Functional enrichment analysis was conducted using ClueGO, a Cytoscape v. 3.10.2 plug-in (http://www.ici.upmc.fr/cluego/, accessed on 3 July 2025), to identify significantly enriched KEGG pathways with a corrected *p*-value < 0.05. Protein–protein interaction network analysis was performed using the STRING v12.0 platform (http://string-db.org), with *Canis lupus familiaris* set as the taxonomy. A medium confidence level (score 0.4) was applied, and only known interactions experimentally determined (pink edges) or based on curated databases (light blue edges) were considered.

## 5. Conclusions

The present study investigated, for the first time, the fecal proteome in two groups of clinically healthy dogs, divided according to breed. The initial hypothesis was that this variable might affect the fecal proteome. Quantitative and qualitative proteomic differences were found by comparing the two groups. The proteins identified as dysregulated in the fecal proteome due to breed differences may suggest new non-invasive biomarkers that need to be investigated in specific studies for monitoring particular aspects of GI chronic inflammatory disease progression, thus facilitating the advancement of new therapeutic strategies in clinical practice.

Although further confirmation of the present findings is needed to understand their potential clinical significance, the present study suggests that breed is a variable that may influence the composition of the canine fecal proteome. More generally, the present results may serve as a useful tool for properly designing future studies that investigate the canine fecal proteome and, in a broader sense, the gastrointestinal environment.

## Figures and Tables

**Table 1:** Qualitative analysis of the proteins present in the two study groups, Weimaraner (WD) and Dalmatian (DD) dogs. Data shown were obtained from pooled samples (n = 13 per group).

N.	Protein Name [OS = *Canis lupus familiaris*]	DD Sequest Score HT	WD Sequest Score HT	Accession	MW [kDa]	Calc. pI
1	Actin, cytoplasmic 1	54.33	129.72	O18840	41.7	5.48
2	Albumin	1077.47	1674.59	P49822	68.6	5.69
3	Aminopeptidase N	1108.5	1540.75	P79143	110.2	6.18
4	Anionic trypsin	302.7	638.78	P06872	26.4	4.83
5	Annexin A2	-	4.33	Q6TEQ7	38.6	7.31
6	Beta-glucuronidase	-	28.99	O18835	74.4	6.57
7	Cadherin-1	481.75	574.79	F1PAA9	97.7	4.81
8	Carboxypeptidase B	213.29	194.1	P55261	47.6	6.6
9	Cathepsin S	-	20.7	Q8HY81	37.2	8.13
10	Serine protease 1	266.92	289.09	P06871	26.2	8.07
11	Chymotrypsin-like elastase fam. member 1	128.43	243.38	Q867B0	27.9	8.46
12	Chymotrypsinogen 2	195.17	172.94	P04813	27.8	7.2
13	Cobalamin binding intrinsic factor	149.5	39.58	Q5XWD5	45	5.78
14	Collagen alpha-5(IV) chain	-	24.04	Q28247	162.1	8.24
15	Cubilin	-	77.81	Q9TU53	397.2	5.44
16	Cytochrome c	165.24	116.71	P00011	11.6	9.58
17	Dihydrolipoyl dehydrogenase, mitochondrial	112.53	162.47	P49819	54.1	7.84
18	Dipeptidyl peptidase 1	240.81	414.86	O97578	49.4	7.03
19	DLA class I histocompatibility antigen, A9/A9 alpha chain	48.96	55.73	P18466	40.4	5.87
20	DLA class II histocompatibility antigen, DR-1 beta chain	89.52	62.49	P18470	30.1	6.15
21	Double-headed protease inhibitor, submandibular gland	26.47	53.34	P01002	12.8	7.93
22	Epididymal secretory glutathione peroxidase	-	3.1	O46607	25.3	8.91
23	Ferritin heavy chain	-	343.06	Q95MP7	21.3	5.88
24	Ferritin light chain	-	165.13	Q53VB8	20.1	6
25	Fibronectin	37.4	94.47	Q28275	243.1	5.99
26	Glyceraldehyde-3-phosphate dehydrogenase	44.69	27.51	Q28259	35.8	8.12
27	Haptoglobin	19.08	252.56	P19006	36.4	6.09
28	Hemoglobin subunit alpha [OS = *Canis latrans*]	-	83.61	P60530	15.4	8.06
29	Hemoglobin subunit beta	-	84.53	P60524	16	8.05
30	Ig heavy chain V region GOM	188.5	258.07	P01784	12.4	5.4
31	Ig heavy chain V region MOO	-	67.5	P01785	12.7	4.72
32	Ig kappa chain V region GOM	87.19	46.27	P01618	12	6.61
33	Keratin, type I cytoskeletal 10	150.23	153.13	Q6EIZ0	57.7	5.15
34	Keratin, type I cytoskeletal 9	9.54	13.88	O18740	76.3	5.95
35	Keratin, type II cytoskeletal 1	148.26	121.34	Q6EIY9	63.8	7.84
36	Keratin, type II cytoskeletal 2 epidermal	43.31	37.37	Q6EIZ1	64.5	7.74
37	Lysozyme C, milk isozyme	39.31	34.15	P81708	14.5	8.29
38	Lysozyme C, spleen isozyme	-	15.41	P81709	14.6	8.81
39	Myosin-2	-	127.86	Q076A7	223.1	5.81
40	Myosin-4	-	175.55	Q076A5	222.9	5.76
41	Myosin-7	-	87.9	P49824	222.8	5.73
42	Myosin-13	-	20.54	Q076A3	223.2	5.68
43	Myosin-16	-	26.91	F1PT61	222.5	6.02
44	NPC intracellular cholesterol transporter 2	48.56	63.39	Q28895	16	8.02
45	Nucleoside diphosphate kinase A	40.31	58.11	Q50KA9	17.2	6.01
46	Nucleoside diphosphate kinase B	50.71	61.42	Q50KA8	17.4	7.99
47	Pancreatic secretory granule membrane major glycoprotein GP2	20.86	68.68	P25291	56.7	5.62
48	Pantetheinase	-	5.39	Q9TSX8	57.4	6.65
49	Phosphatidylethanolamine-binding protein 1	107.81	58.74	Q3YIX4	20.9	7.49
50	Proteasome subunit beta type-8	-	8.11	Q5W416	30.5	7.72
51	Sodium/calcium exchanger 1	-	28.99	P23685	107.9	4.96
52	Superoxide dismutase [Cu-Zn]	306.13	291.48	Q8WNN6	15.9	6.11
53	Tissue alpha-L-fucosidase	51.38	253.61	P48300	53.7	6.74
54	Trefoil factor 2	62.11	64.23	Q863J2	14.1	7.65
55	Trefoil factor 3	187.7	203.52	Q863B4	8.9	4.94
56	Triosephosphate isomerase	221.29	162.87	P54714	26.7	7.33
57	Ubiquitin-60S ribosomal protein L40	29.11	33.4	P63050	14.7	9.83
58	WAP four-disulfide core domain protein 2	47.9	-	A0A8I3NWP8	63.8	7.84
		**Tot. 38**	**Tot. 57**			

**Table 2:** Proteins with different abundance levels in the feces of Dalmatian dogs compared to Weimaraner dogs. In green, proteins that are more abundant in DDs, and in red, proteins that are more abundant in WDs. Data shown were obtained from pooled samples (n = 13 per group).

Gene Name	Protein Name	Protein ID	Sequence Coverage	Score Sequest HT	N. Peptides	Fold Change (DD/WD)	*p*-Value
MYH16	Myosin-16	F1PT61	2	36.66	3	7.80	0.0000
CBLIF	Cobalamin binding intrinsic factor	Q5XWD5	24	189.08	9	3.58	0.0008
	Ig kappa chain V region GOM	P01618	34	131.02	2	3.51	0.0008
CPB1	Carboxypeptidase B	P55261	42	405.24	14	2.02	0.0105
TPI1	Triosephosphate isomerase	P54714	78	384.16	14	1.90	0.0118
GAPDH	Glyceraldehyde-3-phosphate dehydrogenase	Q28259	24	72.2	5	1.78	0.0298
PRSS1	Serine protease 1	P06871	81	556.01	12	1.74	0.0342
UBA52	Ubiquitin-60S ribosomal protein L40	P63050	27	54.1	3	1.56	0.0209
LYZ	Lysozyme C, spleen isozyme	P81709	38	15.41	3	0.67	0.0321
NPC2	NPC intracellular cholesterol transporter 2	Q28895	42	111.95	5	0.60	0.0223
PRSS2	Anionic trypsin	P06872	60	969.67	8	0.55	0.0264
VNN1	Pantetheinase	Q9TSX8	5	5.39	2	0.55	0.0013
HBB	Hemoglobin subunit beta	P60524	66	88.01	11	0.32	0.0007
HBA	Hemoglobin subunit alpha	P60530	62	94.38	6	0.23	0.0121
FTH1	Ferritin heavy chain	Q95MP7	85	348.38	16	0.14	0.0535

**Table 3:** The KEGG (Kyoto Encyclopedia of Genes and Genomes) pathway analysis of dogs (*Canis lupus familiaris*) in the two different breed groups. The graphical representation of these pathways is shown in Figure 5a.

ID	Term	Term *p*-Value Corrected with Bonferroni Step Down	Group *p*-Value Corrected with Bonferroni Step Down	% Associated Genes	Nr. Genes	Associated Genes
KEGG:00010	Glycolysis/gluconeogenesis	0.0075	0.0050	2.30	2	GAPDH, TPI1
KEGG:04972	Pancreatic secretion	0.0055	0.0094	2.20	2	CPB1, PRSS2
KEGG:04974	Protein digestion and absorption	0.0033	0.0094	2.00	2	CPB1, PRSS2

**Table 4:** Dogs included in this study.

Variable	Weimaraner Dogs (WDs, n = 13)	Dalmatian Dogs (DDs, n = 13)
Clinical Health Status	Healthy	Healthy
Medication Status	No medications	No medications
Diarrhea (last month)	None reported	None reported
Other Clinical Signs (last month)	None reported	None reported
Parasite Treatments	Regular treatments	Regular treatments
Diet	Maintenance kibble	Maintenance kibble
Percentage of Male/Female Dogs	46%/54	38%/62%
Age (mean ± SD, years)	5.5 ± 2.9	7.5 ± 3.7

## Data Availability

The data presented in this study are contained within this article or in the Supplementary Materials.

## Supplementary Materials

The following supporting information can be downloaded at https://www.mdpi.com/article/10.3390/ijms26178247/s1.

## References

[1] Cerquetella M., Rossi G., Spaterna A., Tesei B., Gavazza A., Pengo G., Pucciarelli S., Scortichini L., Sagratini G., Ricciutelli M. (2019). Fecal Proteomic Analysis in Healthy Dogs and in Dogs Suffering from Food Responsive Diarrhea. Sci. World J..

[2] Cerquetella M., Marchegiani A., Mangiaterra S., Rossi G., Gavazza A., Tesei B., Spaterna A., Sagratini G., Ricciutelli M., Polzonetti V. (2021). Faecal proteome in clinically healthy dogs and cats: Findings in pooled faeces from 10 cats and 10 dogs. Vet. Rec. Open.

[3] Rossi G., Gavazza A., Vincenzetti S., Mangiaterra S., Galosi L., Marchegiani A., Pengo G., Sagratini G., Ricciutelli M., Cerquetella M. (2021). Clinicopathological and Fecal Proteome Evaluations in 16 Dogs Presenting Chronic Diarrhea Associated with Lymphangiectasia. Vet. Sci..

[4] O’Reilly E.L., Horvatić A., Kuleš J., Gelemanović A., Mrljak V., Huang Y., Brady N., Chadwick C.C., Eckersall P.D., Ridyard A. (2022). Faecal proteomics in the identification of biomarkers to differentiate canine chronic enteropathies. J. Proteomics.

[5] Cerquetella M., Mangiaterra S., Rossi G., Gavazza A., Marchegiani A., Sagratini G., Ricciutelli M., Angeloni S., Fioretti L., Marini C. (2023). Fecal Protein Profile in Eight Dogs Suffering from Acute Uncomplicated Diarrhea before and after Treatment. Vet. Sci..

[6] Cerquetella M., Mangiaterra S., Pinnella F., Rossi G., Marchegiani A., Gavazza A., Serri E., Di Cerbo A., Marini C., Cecconi D. (2023). Fecal Proteome Profile in Dogs Suffering from Different Hepatobiliary Disorders and Comparison with Controls. Animals.

[7] Heilmann R.M., Steiner J.M. (2018). Clinical utility of currently available biomarkers in inflammatory enteropathies of dogs. J. Vet. Intern. Med..

[8] Marsilio S., Dröes F.C., Dangott L., Chow B., Hill S., Ackermann M., Estep J.S., Lidbury J.A., Suchodolski J.S., Steiner J.M. (2021). Characterization of the intestinal mucosal proteome in cats with inflammatory bowel disease and alimentary small cell lymphoma. J. Vet. Intern. Med..

[9] Albasan H., Lulich J.P., Osborne C.A., Lekcharoensuk C. (2005). Evaluation of the association between sex and risk of forming urate uroliths in Dalmatians. J. Am. Vet. Med. Assoc..

[10] Craven M., Simpson J.W., Ridyard A.E., Chandler M.L. (2004). Canine inflammatory bowel disease: Retrospective analysis of diagnosis and outcome in 80 cases (1995–2002). J. Small Anim. Pract..

[11] Kathrani A., Werling D., Allenspach K. (2011). Canine breeds at high risk of developing inflammatory bowel disease in the south-eastern UK. Vet. Rec..

[12] Lehmann T., Schallert K., Vilchez-Vargas R., Benndorf D., Püttker S., Sydor S., Schulz C., Bechmann L., Canbay A., Heidrich B. (2019). Metaproteomics of fecal samples of Crohn’s disease and Ulcerative Colitis. J. Proteomics.

[13] McConnell R.E., Benesh A.E., Mao S., Tabb D.L., Tyska M.J. (2011). Proteomic analysis of the enterocyte brush border. Am. J. Physiol. Gastrointest. Liver Physiol..

[14] Engevik M.A., Engevik A.C. (2022). Myosins and membrane trafficking in intestinal brush border assembly. Curr. Opin. Cell Biol..

[15] Davidson G.P., Cutz E., Hamilton J.R., Gall D.G. (1978). Familial enteropathy: A syndrome of protracted diarrhea from birth, failure to thrive, and hypoplastic villus atrophy. Gastroenterology.

[16] VanDussen K.L., Stojmirović A., Li K., Liu T.C., Kimes P.K., Muegge B.D., Simpson K.F., Ciorba M.A., Perrigoue J.G., Friedman J.R. (2018). Abnormal Small Intestinal Epithelial Microvilli in Patients With Crohn’s Disease. Gastroenterology.

[17] Yokoyama M., Kimura M.Y., Ito T., Hayashizaki K., Endo Y., Wang Y., Yagi R., Nakagawa T., Kato N., Matsubara H. (2021). Myosin Light Chain 9/12 Regulates the Pathogenesis of Inflammatory Bowel Disease. Front. Immunol..

[18] Heybrock S., Kanerva K., Meng Y., Ing C., Liang A., Xiong Z.J., Weng X., Ah Kim Y., Collins R., Trimble W. (2019). Lysosomal integral membrane protein-2 (LIMP-2/SCARB2) is involved in lysosomal cholesterol export. Nat. Commun..

[19] Sturley S.L., Patterson M.C., Balch W., Liscum L. (2004). The pathophysiology and mechanisms of NP-C disease. Biochim. Biophys. Acta.

[20] Larabi A., Barnich N., Nguyen H.T.T. (2020). New insights into the interplay between autophagy, gut microbiota and inflammatory responses in IBD. Autophagy.

[21] Du X., Kumar J., Ferguson C., Schulz T.A., Ong Y.S., Hong W., Prinz W.A., Parton R.G., Brown A.J., Yang H. (2011). A role for oxysterol-binding protein-related protein 5 in endosomal cholesterol trafficking. J. Cell Biol..

[22] Garver W.S., Krishnan K., Gallagos J.R., Michikawa M., Francis G.A., Heidenreich R.A. (2002). Niemann-Pick C1 protein regulates cholesterol transport to the trans-Golgi network and plasma membrane caveolae. J. Lipid Res..

[23] Nitto T., Onodera K. (2013). Linkage between coenzyme a metabolism and inflammation: Roles of pantetheinase. J. Pharmacol. Sci..

[24] van Diepen J.A., Jansen P.A., Ballak D.B., Hijmans A., Hooiveld G.J., Rommelaere S., Galland F., Naquet P., Rutjes F.P., Mensink R.P. (2014). PPAR-alpha dependent regulation of vanin-1 mediates hepatic lipid metabolism. J. Hepatol..

[25] Giessner C., Millet V., Mostert K.J., Gensollen T., Vu Manh T.P., Garibal M., Dieme B., Attaf-Bouabdallah N., Chasson L., Brouilly N. (2018). Vnn1 pantetheinase limits the Warburg effect and sarcoma growth by rescuing mitochondrial activity. Life Sci. Alliance.

[26] Casazza A., Di Conza G., Wenes M., Finisguerra V., Deschoemaeker S., Mazzone M. (2014). Tumor stroma: A complexity dictated by the hypoxic tumor microenvironment. Oncogene.

[27] Millet V., Gensollen T., Maltese M., Serrero M., Lesavre N., Bourges C., Pitaval C., Cadra S., Chasson L., Vu Man T.P. (2023). Harnessing the Vnn1 pantetheinase pathway boosts short chain fatty acids production and mucosal protection in colitis. Gut.

[28] Wei J., Tao G., Xu B., Wang K., Liu J., Chen C.H., Dunn J.C.Y., Currie C., Framroze B., Sylvester K.G. (2022). Soluble Protein Hydrolysate Ameliorates Gastrointestinal Inflammation and Injury in 2,4,6-Trinitrobenzene Sulfonic Acid-Induced Colitis in Mice. Biomolecules.

[29] Harder B.J., Lekkerkerker A.N., Casavant E.P., Hackney J.A., Nguyen A., McBride J.M., Mathews W.R., Anania V.G. (2024). Comprehensive profiling of the human fecal proteome from IBD patients with DIA-MS enables evaluation of disease-relevant proteins. Proteomics Clin. Appl..

[30] Jablaoui A., Kriaa A., Mkaouar H., Akermi N., Soussou S., Wysocka M., Wołoszyn D., Amouri A., Gargouri A., Maguin E. (2020). Fecal Serine Protease Profiling in Inflammatory Bowel Diseases. Front. Cell Infect. Microbiol..

[31] Al-Awami H.M., Raja A., Soos M.P. (2024). Physiology, gastric intrinsic factor. StatPearls [Internet].

[32] Toresson L., Steiner J.M., Suchodolski J.S., Spillmann T. (2016). Oral Cobalamin Supplementation in Dogs with Chronic Enteropathies and Hypocobalaminemia. J. Vet. Intern. Med..

[33] Mosnier L.O., Bouma B.N. (2006). Regulation of fibrinolysis by thrombin activatable fibrinolysis inhibitor, an unstable carboxypeptidase B that unites the pathways of coagulation and fibrinolysis. Arterioscler. Thromb. Vasc. Biol..

[34] Gu X., Dai X., Huang Y., Zhang Y., Dong L., Gao C., Wang F. (2023). Differential roles of highly expressed PFKFB4 in colon adenocarcinoma patients. Sci. Rep..

[35] Mangiaterra S., Vincenzetti S., Rossi G., Marchegiani A., Gavazza A., Petit T., Sagratini G., Ricciutelli M., Cerquetella M. (2022). Evaluation of the Fecal Proteome in Healthy and Diseased Cheetahs (*Acinonyx jubatus*) Suffering from Gastrointestinal Disorders. Animals.

[36] Bradford M.M. (1976). A rapid and sensitive method for the quantitation of microgram quantities of protein utilizing the principle of protein-dye binding. Anal. Biochem..

[37] Perez-Riverol Y., Bandla C., Kundu D.J., Kamatchinathan S., Bai J., Hewapathirana S., John N.S., Prakash A., Walzer M., Wang S. (2025). The PRIDE database at 20 years: 2025 update. Nucleic Acids Res..

[38] Di Carlo C., Sousa B.C., Manfredi M., Brandi J., Dalla Pozza E., Marengo E., Palmieri M., Dando I., Wakelam M.J.O., Lopez-Clavijo A.F. (2021). Integrated lipidomics and proteomics reveal cardiolipin alterations, upregulation of HADHA and long chain fatty acids in pancreatic cancer stem cells. Sci. Rep..

